# Editorial: Molecular mechanisms of osteoporosis

**DOI:** 10.3389/fendo.2023.1301244

**Published:** 2023-10-20

**Authors:** Ziqing Li, Kenneth Guangpu Yang

**Affiliations:** ^1^ Department of Joint Surgery, Shandong Provincial Hospital Affiliated to Shandong First Medical University, Jinan, Shandong, China; ^2^ Orthopaedic Research Laboratory, Medical Science and Technology Innovation Center, Shandong First Medical University & Shandong Academy of Medical Sciences, Jinan, Shandong, China; ^3^ Department of Orthopaedics and Traumatology, The Chinese University of Hong Kong, Hong Kong, Hong Kong SAR, China

**Keywords:** osteoporosis, bone remodeling, antiosteoporotic medications, drug targets and nanomaterials, bioinformatics

## Introduction

Osteoporosis is a metabolic bone disorder that threatens holistic well-being and economic stability worldwide due to its high prevalence in the elderly. Osteoporosis is associated with systemic reduction in bone mass and microarchitectural deterioration of bone tissue causing an increased risk of bone fracture. Osteoporosis together with other age-related conditions disturbs the pace and process by which osteoclasts (OCs) and osteoblasts (OBs) activities are coupled and balanced, and therefore normal bone remodeling and calcium homeostasis are deranged. This collection of seven papers constitutes the Research Topic and brings together vital studies investigating osteoporosis’s molecular mechanisms and related complications, and the novel advances in drug development targeting functional genes/proteins involved in the pathology of osteoporosis.

## Molecular studies of functional genes in osteoporosis

A total of four articles are included in this part to discuss the recent advancements in functional genes in osteoporosis. These studies aimed to establish a foundation for translating insights from human genetic research into innovative treatment solutions for osteoporosis. Recent studies suggest that SIRT3 contributes to the physiological processes associated with the senescence of bone marrow mesenchymal stem cells (BMSCs) and the differentiation of BMSCs and osteoclasts. Nevertheless, the exact effects and mechanisms underlying SIRT3’s role in osteoporosis remain unclear. Hu and Wang reviewed the multifaceted role of SIRT3 in osteoporosis, and they elaborated on the cell types involved in bone remodeling and thereby built a theoretical basis for SIRT3 being a therapeutic target for osteoporosis. In another article, Yang et al. studied the role of p.Ser267Phe mutation in NTCP deficiency and its subsequent long-term impacts. Their study offered insightful revelations and underscored the value of genetic research in unraveling novel and potential therapeutic approaches. Yang et al. revealed that NTCP deficiency, caused by the p.Ser267Phe mutation in the SLC10A1 gene, affected the circulating level of bile acid leading to vitamin D deficiency, bone loss, and gallbladder abnormalities. Therefore, regular monitoring on level of bile acids, amount of vitamin D storage, and bone density in those with NTCP deficiency was recommended. Research advances on WNT1 and PLS3 mutations by Loid et al. demonstrated the association between plasma lipocalin-2 level and fibroblast growth factor-23 (FGF23). This association could be linked to abnormal WNT1 and PLS3 signaling which indicated its significant role in bone metabolism. This study revealed that lipocalin-2 was not altered in WNT1 or PLS3 mutation-positive subjects, while it was associated with C-terminal and intact FGF23 in WNT1 and PLS3 patients respectively. This finding indicates a possible role of lipocalin-2 in the FGF23-mediated signaling pathway in the osteoporosis caused by these mutations. This study provides novel insights into targeted therapeutics in osteoporosis. The correlation of lipocalin-2 with serum transferrin in WNT1 potentially outlines future areas of exploration regarding the role of iron status in abnormal WNT1 signaling. Zhao et al. used robust methods to identify hub genes that interact strongly during osteoporosis progression, including gene co-expression network analysis (WGCNA) and differential analysis on gene expression profiles from the GEO database. Their meticulously conducted study uncovered significant insights into the role oxidative stress plays in osteoporosis, alongside a potentially promising treatment development strategy targeting the MAPKAPK2 proteins, such as PPP1R15A, CYB5R3, BCL2L1, and MAPKAPK2. These markers provide further insights into the role of oxidative stress in osteoporosis development and potential treatment targets.

## Studies of pharmaceutical intervention in osteoporosis

A total of three articles about advancements in disease treatment for osteoporosis are included in this part. Zhang et al. highlighted the potential therapeutic use of berbamine in treating postmenopausal osteoporosis (PMOP). Their results showed that berbamine inhibited bone resorption in PMOP mice and offered a fascinating spotlight on this traditional Chinese medicinal plant with its potential in treating diseases beyond its current clinical utilization for leukopenia. The detailed depiction of berbamine’s inhibitory role against RANKL-induced osteoclastogenesis denoted a promising shift in osteoporosis treatment. Another novel study by Che et al. unraveled a new dimension in the treatment of postmenopausal osteoporosis with FDA-approved PTH 1-34. The research delved into the less-explored area of iron metabolism and studied its role in the regulation of unloading-induced osteoporosis. Their study documented implications of iron overload as a significant risk factor for bone loss which enhanced the current understanding of osteoporosis and paved the way for future novel therapeutic strategies. By illuminating how PTH 1-34 moderates the imbalance of iron metabolism in unloading mice via Nrf2 activation and subsequent increase in hepatic hepcidin expression, Che et al. provided compelling evidence supporting the osteoprotective effect of PTH 1-34 beyond its primary mechanism of action. On top of that, their results also indicated that Nrf2 inhibitors might have an unanticipated adverse effect on the benefits of PTH 1-34 treatment. Kulkarni et al. offered a new perspective on the bone-conserving properties of the plant Coleus forskohlii root extract (CFE) which is typically known for its valuable role in preventing weight gain amongst obese individuals. Their study suggested that forskolin consequently fostered an osteogenic effect. Forskolin is the predominant compound present in CFE and is notable as an adenylate cyclase (AC) activator that raises intracellular cyclic adenosine monophosphate (cAMP) levels in osteoblasts. Kulkarni et al. utilized an examination in adult ovariectomized (OVX) rats and demonstrated how CFE sustains bone mass and microarchitecture to levels comparable to sham-operated rats. Intriguingly, the surface-referent bone formation is significantly increased in CFE-treated rats, amidst several other vital findings pertaining to the preservation of bone mass, microarchitecture, and strength.

In conclusion, potential therapeutics, from plant extracts to FDA-approved drugs and novel genetic targets, are revealing molecules and pathways for preventing and treating osteoporosis. Ongoing research continues to elucidate molecular and cellular mechanisms, paving the way for the discovery and optimization of future treatments for this prevalent disease.

## Author contributions

ZL: Writing – original draft, Writing – review & editing. KY: Writing – original draft, Writing – review & editing.

